# Heterogeneity of the rearing environment enhances diversity of microbial communities in intensive farming

**DOI:** 10.1186/s42523-024-00359-8

**Published:** 2024-12-20

**Authors:** Roghaieh Ashrafi, Lotta-Riina Sundberg, Pekka Hyvärinen, Anssi Karvonen

**Affiliations:** 1 Department of Biological and Environmental Science, University of Jyväskylä, Jyväskylä, Finland; 2 Department of Biological and Environmental Science and Nanoscience Center, University of Jyväskylä, Jyväskylä, Finland; 3 Aquatic Population Dynamics, Natural Resources Institute Finland (Luke), Paltamo, Finland

**Keywords:** Enriched rearing, Microbiome, Atlantic salmon, Intensive farming, Biofilm, Microbial composition

## Abstract

Heterogeneity of the rearing environment in farmed animals can improve welfare and stocking success by enhancing natural behaviours, reducing stress, and decreasing pathogen occurrence. Although microbial diversity is often associated with well-being, their direct and indirect effects on health of farmed animals remain underexplored. We examined the impact of structural heterogeneity of aquaculture tanks on microbial communities in tank biofilm and fish gut microbiome. Enrichment (stones and shelters) significantly promoted microbial diversity and community homogeneity in tank biofilm. However, diversity of gut microbiome did not depend on rearing treatment or microbial composition of the environment. Fish in enriched tanks exhibited greater compositional variation in gut microbiome than those in standard tanks. Tanks without enrichments had higher occurrence of potentially pathogenic bacterial families (*Corynebacteriaceae* and *Staphylococcaceae*), while enriched tanks had more beneficial gut microbes (*Lactobacillus*). Microbial diversity in tank biofilm was negatively associated with fish mortality during a natural epidemic of *Flavobacterium columnare*, suggesting a protective effect of diverse microbial communities. These findings support environmental enrichment in mitigating disease outbreaks through enhanced microbial diversity, providing important implications for disease control and sustainable health management in aquaculture.

**Supplementary Information:**

The online version contains supplementary material available at 10.1186/s42523-024-00359-8.

## Background

Intensive animal farming is often associated with rapid spread and high severity of diseases, as well as with emergence of novel pathogens [25, 63, 71, 75]. One of the greatest challenges in development and sustainability of industrial aquaculture is the management of infectious diseases, which cause direct production losses and increased operating costs as pathogens and parasites thrive in high host densities [44, 63, 78]. Conventionally, antimicrobials and other chemicals have been used to treat infections. This can be costly and often associated with secondary effects such as development of drug-resistant pathogens and release of chemical residues into the environment [20, 62, 65]. Consequently, strategies for controlling infections that are both economically and environmentally sustainable are in great demand.

Aquaculture tanks typically represent highly simplified environments compared to the wild, which can induce behavioral and physiological stress reactions in fish and make them more susceptible to diseases through suppression of the immune system [6, 70]. Therefore, environmental enrichment has been used to increase heterogeneity of the rearing environment and subsequently improve physical and social well-being of farmed animals [49, 54, 56]. In fish, enrichment of the rearing environment has been shown to improve foraging behavior, exploration, learning capacities, neuronal plasticity and post-release survival [11, 22, 24, 64, 88, 89]. Fish reared in structured environments have also been shown to have higher growth, reduced aggression and lower cortisol level, which suggests lower stress associated with enriched environment [57]. There is also evidence supporting the use of natural materials, such as stones, in attenuating disease progression and mortality in different species and populations of aquaculture fish [28, 66]. However, detailed mechanisms underlying the positive effects of enriched rearing on fish welfare are still largely unknown.

Microbiota inside and outside the hosts has recently been shown to significantly impact health [7, 46]. Microbes can maintain host immune homeostasis, and changes in the diversity and composition of the microbial community can either promote or mitigate disease occurrence [16, 21]. Microbial communities in the surrounding environment can also influence health through ecological interactions between the microbes [4, 15, 74]. For example, environmental microbial communities can limit occurrence and epidemics of pathogens through direct interactions such as nutrient competition, niche exclusion, toxic substances and metabolite production [7, 15]. Similar effects could also emerge indirectly if microbes improved host nutritional status and growth, or degraded toxic compounds [60, 61]. In the aquaculture realm, additional structures used in enriched rearing could potentially alter diversity and composition of microbial communities in tanks and in fish, thereby reducing disease outbreaks [66]. Details of microbial community structure associated with different aquaculture rearing methods and the potential role they play in disease epidemiology, however, remain largely unexplored.

Here, we investigated how structural heterogeneity of aquaculture tanks affected the diversity and composition of microbial communities in tanks and fish in moderate production scale fish densities. We focused on tank biofilm and fish gut microbiome and conducted a sampling campaign on enriched (tanks with stones, covers and changes in water inflow) and standard (no additional structures or changing water flow dynamics) tanks. We also examined the relationship between microbial diversity and fish mortality rate during a natural outbreak of a pathogenic bacterium, *Flavobacterium columnare*. We found that enriched tank housed more diverse and more homogeneous microbial biofilms, but more heterogeneous gut microbiomes. We also found suggestive evidence of a negative relationship between biofilm microbial diversity and mortality of fish during the bacterial outbreak. Overall, the present results suggest that diversity of microbial communities can be enhanced by altering conventional rearing practices, which may also play a role in epidemiology of aquatic diseases.

## Materials and methods

### Fish origin and tank setup

Three populations of anadromous brown trout (*Salmo trutta*), initially originating from different, geographically isolated rivers (River Ingarskilanjoki, Isojoki, and Lestijoki), were used [22, 66]. The fish were raised in Kainuu Fisheries Research Station (www.kfrs.fi, 64.404°N, 27.516°E), which is a flow-through facility taking water from the nearby Lake Kivesjärvi. Thus, the water temperature followed that of the lake and was the same for all tanks. The fish were brought into the facility at eye‐egg stage in end of March and were divided into 3.2 m^2^ tanks, using six replicate tanks for each population (total 18 tanks) with 1950 fish in each tank (Table S1). Half of the tanks of each population were equipped with environmental enrichments including gravel and shelters [29, 66] (Fig. S1). The direction of the water inflow was also changed regularly. The other half remained as standard tanks without enrichment and water inflow change (Table S1). Tanks were cleaned regularly according to normal aquaculture protocols and the fish were fed with automated feeders containing commercial fish feed (Veronesi VITA 0.2/0.5 and Inicio Plus G 0.4).

Fish were maintained in these tanks until end of August, except for the population Ingarskilanjoki (6 tanks), which was transferred to another experiment in early August, after sampling of the microbial communities in July (see below). The remaining 12 tanks, housing the populations Isojoki and Lestijoki, experienced an outbreak of the bacterium *Flavobacterium columnare* in the second half of August, which was subsequently treated with a course of antibiotics. The number of dead fish was recorded daily during the entire experimental period in March-August. Bacterial cultivations from gills of a sample of dead fish were made on Shieh agar supplemented with tobramycin to confirm the presence of *F. columnare* [13].

### Sample collection

Tank biofilm samples were collected using a microscope glass slide installed inside an open-ended falcon tube sealed with plastic netting at both ends to prevent fish entering the tube. Two tubes were anchored on opposite sides of each tank for 14 days in July. Slides were then retrieved and stored in 95% ethanol prior to DNA extraction. Cells were harvested by scraping the slides into bead beating tubes. To examine the microbiota of fish gut, five fish (age approx. 4 months) were randomly sampled from each of the 18 tanks in end of July, euthanized using benzocaine solution and measured for total length and mass. Entire gut was dissected from each fish, preserved in 95% ethanol, and directly stored at − 20 °C.

### DNA isolation, amplification, and sequencing

To ensure optimal recovery of cell from stored samples, samples were first centrifuged at 13,000*g for 10 min to pellet all cells, after which the ethanol was carefully removed, and the remaining solid material was transferred to bead beating tubes. DNA was isolated from both gut and biofilm samples using the Powersoil® DNA kit (MoBio Laboratories, Carlsbad, CA, USA) according to the manufacturer’s instructions. Isolated DNA was stored at − 20 °C until further use. DNA concentration was quantified with Qubit fluorometer (Qiagen) to normalize the DNA concentrations to 10 ng template DNA for samples. The V4 region of the 16S rRNA gene was amplified by PCR in a triplicate reaction using Illumina tagged primers: 515F and 806R.

A touchdown PCR was performed for all samples to avoid co-amplification of host DNA. This used 10 μl of Phusion 2 × master mix, 1 μl (5 μM) of each specific primer, 8 μl of sterile nuclease-free water, and 1 μl of DNA (around 10 ng/μL). Touch-down PCR was set up as follows: initial denaturation step at 98 °C for 30 s followed by 12 cycles (1 °C decrease of annealing temperature per cycle) of 62 °C, 10 s; and 72 °C for 45 s. After the initial touchdown PCR cycles, an additional 21 cycles were run at 98 °C for 15 s (denaturation), 56 °C for 30 s (annealing) and 72 °C for 30 s (extension), and a final extension of 72 °C for 10 min. Amplification of each sample was performed in triplicate and combined to a final volume of 75 μL. The PCR products were analyzed by electrophoresis in 1.5% (w/v) agarose gels. Library preparations and Illumina sequencing using 2 × 300 bp v2 chemistry were performed by Molecular Medicine Finland Institute (FIMM, University of Helsinki: www.fimm.fi). One extraction blank was prepared and sequenced together with the samples to assess the degree of background microbial contamination. In addition, a mock community (ZymoBIOMICS Microbial Community DNA Standard, Zymo Research) was amplified and sequenced.

### Sequence data processing

Sequencing data were analyzed using Mothur (v.1.40.2, www.mothur.org) according to the Mothur Illumina Miseq standard operating procedure (SOP) [68]. Briefly, after forming contigs, primers were trimmed using PRINSEQ [69] in chipster [26, 27]. The PRINSEQ trimmed sequences were used for the first ‘screen.seqs’ command in Mothur to remove ambiguous sequences and sequences containing homopolymers longer than 6 bp. In addition, any sequences longer than 300 bp were removed. Unique reads (‘unique.seqs’) were aligned (‘align.seqs’) using the Silva bacterial database ‘silva.nr_v138.align’ with flip parameter set to true. Any sequences outside the expected alignment coordinates were removed. The correctly aligned sequences were subsequently filtered (‘filter.- seqs’) with ‘vertical = T’ and ‘trump = ’. The filtered sequences were de-noised by allowing three mismatches in the “pre.clustering” step and chimaeras were removed using Uchime with the dereplicate option set to ‘true’. The chimaera-free sequences were classified using the Silva reference database ‘silva.nr_v138.align’ and the Silva taxonomy database ‘silva.nr_v138.tax’ and a cut-off value of 80%. Contigs were clustered into operational taxonomic units (OTUs) using mothur, based on 97% sequence similarity. Chloroplast, mitochondria, unknown, and eukaryota sequences were removed. Furthermore, Pseudomonas, Halomonas, and Shewanella displayed a considerable abundance in negative controls, exceeding a count of 50,000 sequences, and were subsequently removed from the analysis. The presence of such OTUs at high frequencies in negative samples with notably low DNA concentrations, gives rise to concerns about their authenticity and potential as contaminants, warranting their exclusion from the samples [12]. These genera have commonly been recognized as prevalent contaminants in microbial studies [33, 35, 42, 50].

### Statistical analysis

To analyze alpha diversity, sequences were rarefied utilizing the 'phyloseq::rarefy_even_depth' function implemented in the ‘phyloseq’ R package [48]. Shannon index (α-diversity) was then computed and plotted using the 'microbiome::alpha()' function of the 'microbiome' R package [38]. Shannon diversity is a measure that considers both richness (the number of different species or operational taxonomic units—OTUs) and evenness (the distribution of abundance among those species) of species within a community. Differences in α-diversity between the treatments were analyzed using linear mixed effects model with the 'lmer' function in the lme4 package [5]. Sample source (tank biofilm/fish gut), fish population (Isojoki, Lestijoki, Ingarskilanjoki), and rearing treatment (enriched/standard) were treated as fixed categorical factors and tank ID as a random effect. To analyze pairwise differences between the factors, Tukey's Honest Significant Difference (HSD) tests were conducted using the emmeans package [41].

To investigate differences in microbial community composition (β-diversity), Bray–Curtis dissimilarity [8] and unweighted uniFrac distance metrics [45] were calculated using the relative microbial abundances after removing singletons. The effects of sample source, fish population and rearing treatment were determined using permutational analysis of variance (PERMANOVA with 9,999 permutations) and adonis2 function of the ‘vegan’ package in R [58]. Tank (TankID) was used as a random factor within the permutation block design, where samples are freely permuted (type = free), and tested using the “Terms”-option, equivalent to type I ANOVA tests on main effects [81]. Pairwise effects of fish population and rearing treatment were tested separately for each sample source using the pairwise.adonis2 function [47]. *P*-values for multiple comparisons were adjusted with the Bonferroni correction. Principal coordinate analysis (PCoA) based on the Bray–Curtis dissimilarity matrix using amp_ordinate was used to visualize the distances between samples (Ampvis2, R package version 2.8.3; https://kasperskytte.github.io/ampvis2/) [2]. Taxonomy plots showing relative abundances were plotted using ggplot2 package microbiomeutilities [73] and the Phyloseq packages in R.

Divergence (heterogeneity) in individual tank biofilm samples and gut samples in relation to their median diversity profile, incorporating the effect of rearing treatment, was analyzed using the ‘microbiome’ R-package (http://microbiome.github.com/microbiome) [38]. Kruskal–Wallis test was applied to compare the inter-group variation in gut and biofilm composition. Furthermore, differences in the proportions of 20 most abundant taxa at the family level were assessed between fish populations and rearing treatments using a linear model with tank_ID as a random effect.

Differences in fish survival during the *F. columnare* outbreak in August 10–31 were analyzed using GLMM with rearing treatment (enriched/standard) and fish population (Isojoki/Lestijoki) as fixed factors, and tank_ID as a random factor. Furthermore, the relationship between fish mortality and the measures of α-diversity of the biofilm (OTU richness and Shannon index) of each tank was analyzed separately for the fish populations using Spearman correlation. All analyses were conducted using R ver. 4.2.1.

## Results

**Fig. 1:**
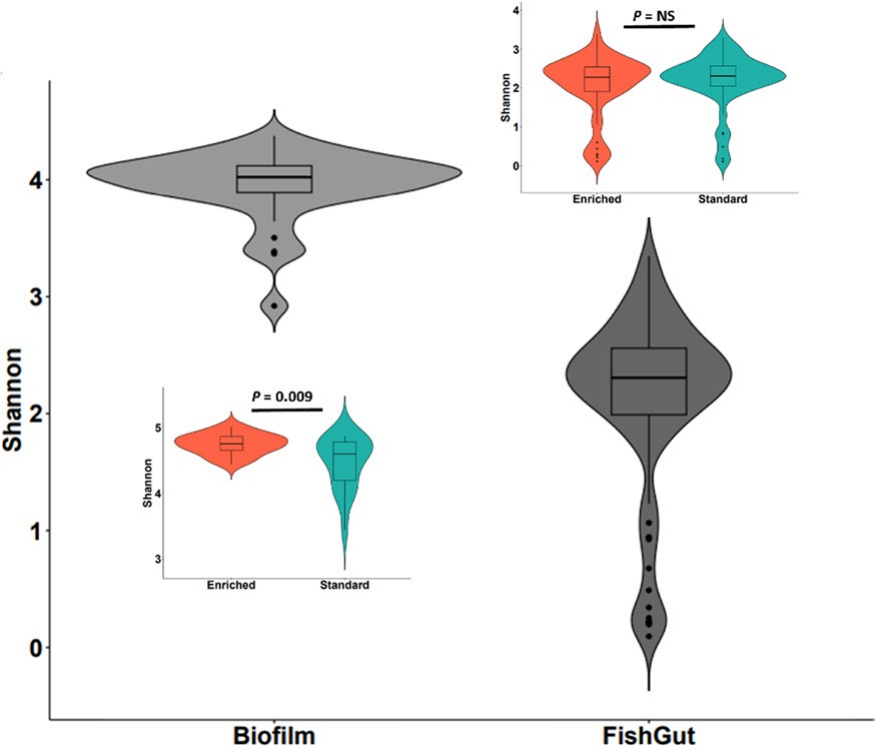
Alpha diversity (Shannon diversity index incorporating both richness and evenness of communities based on OTU-level analysis) in tank biofilm and gut microbiome of brown trout (*Salmo trutta*), and in enriched (orange color) and standard tanks (green color) within each sample source

Our aim was to study how structural heterogeneity (i.e. addition of stones) in aquaculture tanks influenced microbial communities in the tank biofilm and gut microbiome of trout by comparing the communities between enriched and standard rearing conditions. Alpha diversity of tank biofilm communities was significantly higher in enriched tanks (LMM, t_Shannon_ = 2.78, *p* = 0.009), but the diversity in the fish gut did not differ between the rearing treatments (LMM, t_Shannon_ = 0.701, *p* = 0.410). The diversity of the tank biofilm was also significantly higher compared to gut microbiome (LMM, t_Shannon_ = 11.2, *p* < 0.001) (Fig. 1). Furthermore, Shannon diversity of the gut communities was lower in Isojoki population compared to Ingarskilanjoki (LMM, t = -2.60, *p* = 0.021) and Lestijoki (LMM, t = -3.0, *p* = 0.012) (Table S1 and Fig. S1).

Composition of the microbial community (beta diversity) was significantly different between tank biofilm and fish gut microbiome (PERMANOVA: Bray Curtis dissimilarity R^2^ = 0.28, *p* = 0.002; UniFrac distance metric R^2^ = 0.31, *p* = 0.002). However, beta diversity did not differ between enriched and standard tanks in tank biofilm (PERMANOVA: Bray Curtis dissimilarity R^2^ = 0.04, *p* = 0.2; UniFrac distance metric R^2^ = 0.03, *p* = 0.29) or gut microbiome (Bray Curtis dissimilarity R^2^ = 0.01, *p* = 0.32; UniFrac distance R^2^ = 0.01, *p* = 0.51) (Fig. S4). Moreover, microbial composition of the tank biofilm tended to differ between the fish populations (Bray Curtis dissimilarity R^2^ = 0.11, *p* = 0.053; UniFrac distance metric R^2^ = 0.12, *p* = 0.052), but this was not observed for fish gut microbiome (Bray Curtis dissimilarity R^2^ = 0.03, *p* = 0.08; UniFrac distance R^2^ = 0.04, *p* = 0.08) (Fig. S5).

**Fig. 2:**
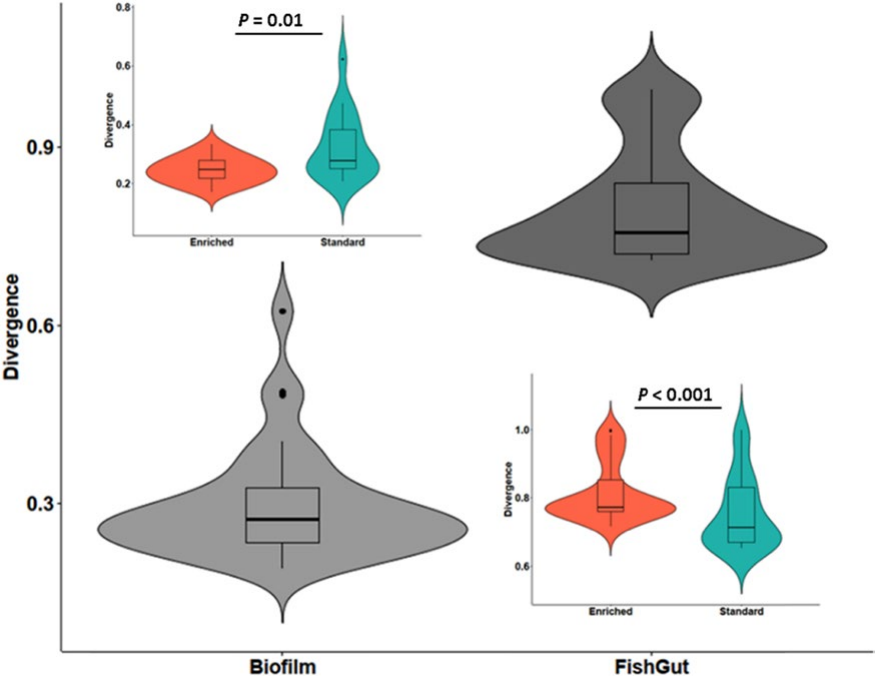
Microbial community divergence in tank biofilm and gut microbiome of brown trout (*Salmo trutta*), and in enriched (orange color) and standard tanks (green color) within each sample source

Microbial communities of the gut samples were more heterogeneous, i.e. showed higher inter-individual variation, compared to biofilm samples between the tanks (KW: χ^2^ = 76.5, *p* < 0.001). Moreover, gut microbial communities exhibited larger inter-individual variation in the enriched tanks compared to standard tanks (KW: χ^2^ = 11.1, p < 0.001), despite their similar overall variance (permutest pseudo-F = 0.7, *p* = 0.3). In contrast, microbial communities of tank biofilm were more uniform in enriched tanks compared to standard tanks (KW: χ^2^ = 5.63, *p* = 0.01) (Fig. 2).

**Fig. 3:**
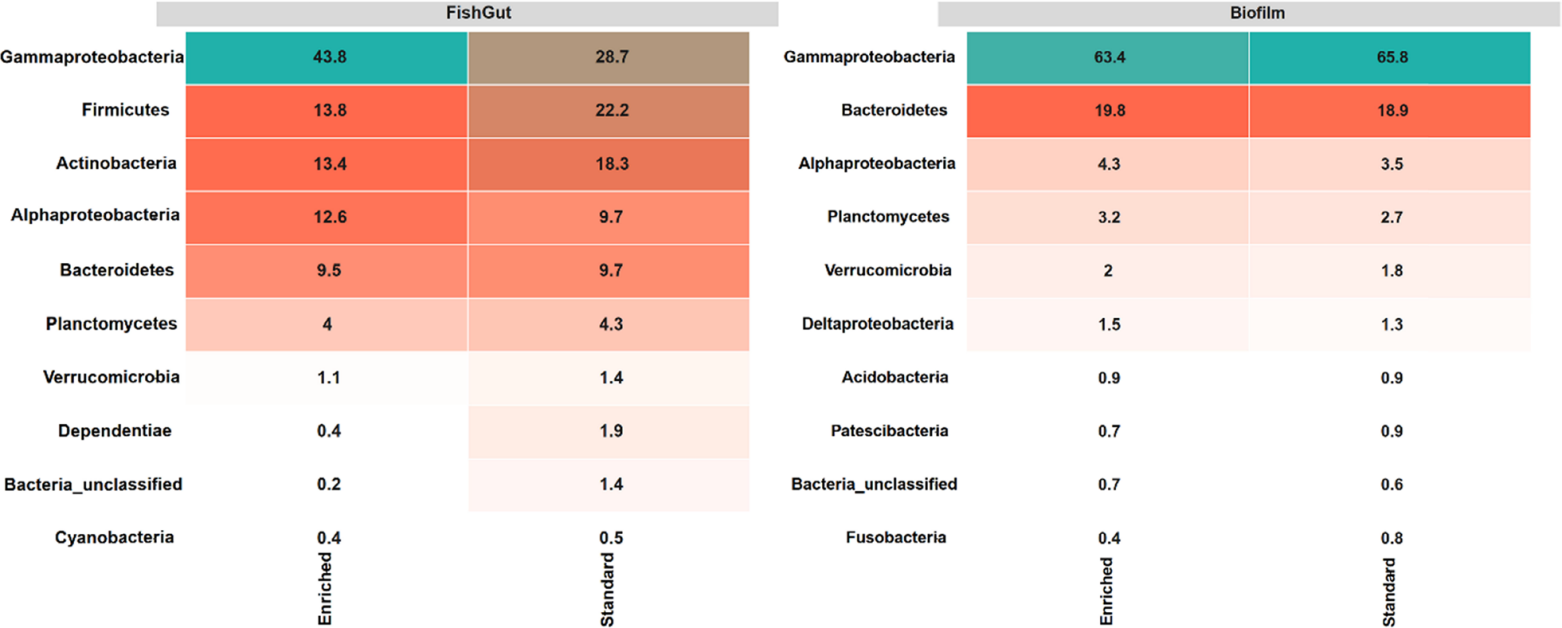
Comparative heatmap illustrating the relative abundance of the top 10 bacterial phyla in tank biofilm and gut microbiome of brown trout (*Salmo trutta*), and in enriched and standard tanks within each sample source. The numerical values within the cells indicate median relative abundance of aggregated operational taxonomic units (OTUs) within each phylum

The dominant microbial phyla in tank biofilm were *Proteobacteria* (68.8%; consisting mainly of *Gammaroteobacteria* 63.5% and *Alphaproteobacteria* 4.1%) and *Bacteroidetes* (19.9%), followed by *Planctomycetes* (3.1%), *Verrucomicrobia* (1.9%) and *Acidobacteria* (0.9%). Within these phyla, the most commonly found families were *Burkholderiaceae* (49.1%), *Flavobacteriaceae* (11.1%), *Steroidobacteraceae* (7.5%), *Chitinophagaceae* (1.5%), and *Rhodobacteraceae* (1.3%) (Fig. 3).

**Fig. 4:**
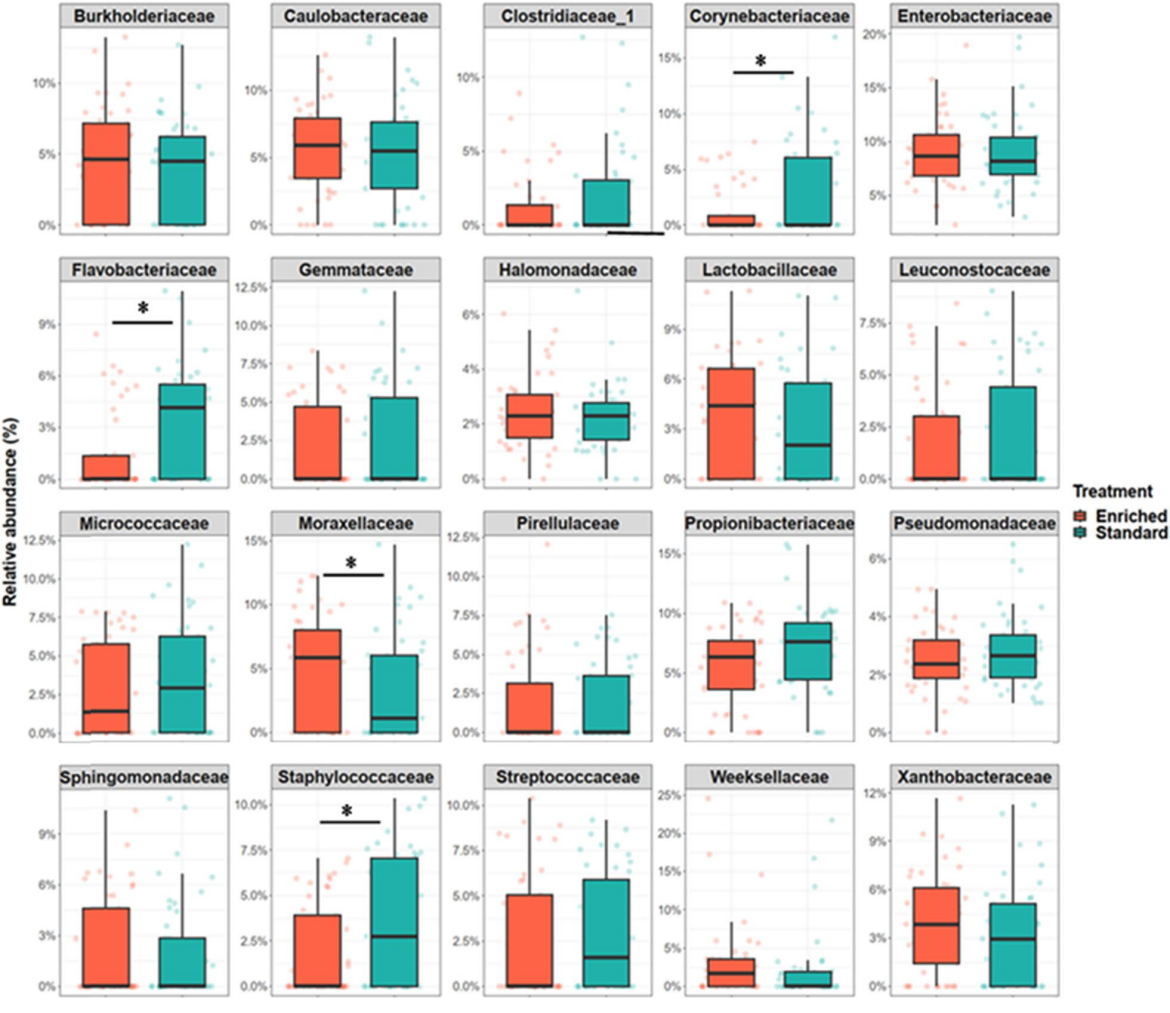
Boxplots (individual samples marked with dots) representing the relative abundance of the 20 most common microbial families in gut microbiome of brown trout (*Salmo trutta*) raised in enriched and standard tanks. Statistically significant differences between the rearing treatments are indicated with asterisk (*p* < 0.05)

In the gut microbiome, *Proteobacteria* (44.9%; consisting mainly of *Gammaproteobacteria* 30.9% and *Alphaproteobacteria* 8.6%), *Firmicutes* (14.4%), *Actinobacteria* (11.4%), and *Bacteroidetes* (2.5%) were the dominant phyla. In tank biofilm, in contrast, *Firmicutes* and *Actinobacteria* had very low relative abundance (≤ 1%). Gut microbiome of fish raised in standard tanks had significantly higher relative abundance of *Corynebacteriaceae* (*t* = 1.96, *p* = 0.04), *Flavobacteriaceae* (*t* = 2.58, *p* = 0.01) and *Staphylococcaceae* (*t* = 2.00, *p* = 0.04). On the other hand, *Moraxellaceae* was more abundant in enriched environment compared to standard (*t* = 1.9, *p* = 0.04) and a similar tendency was observed in *Lactobacillaceae* although this difference was not statistically significant (Fig. 4).

**Fig. 5:**
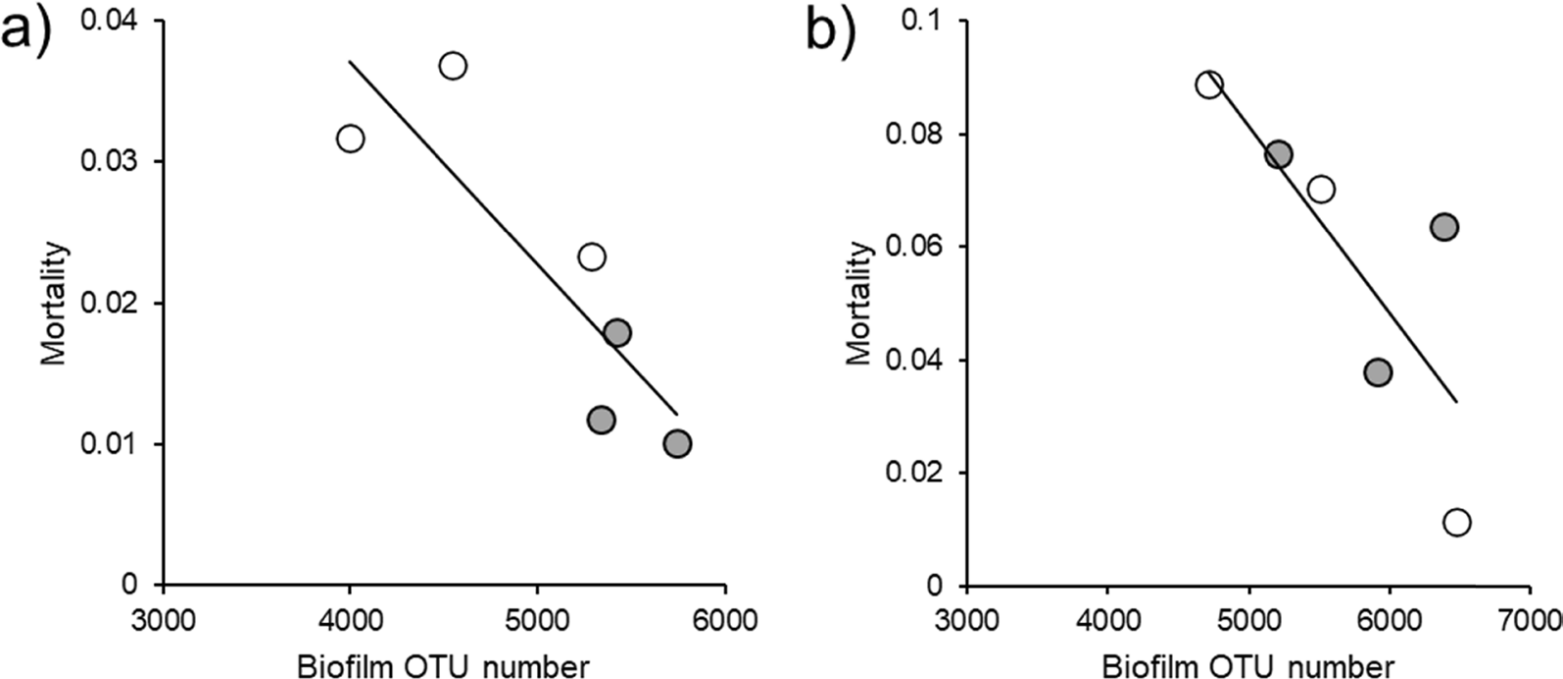
Relationship between biofilm OTU number in enriched (filled symbols) and standard (open symbols) tanks, and mortality during the natural outbreak of the pathogenic bacterium *Flavobacterium columnare* in brown trout originating from **a** Isojoki and **b** Lestijoki population. The fitted line is linear regression to indicate direction of the relationship

Sixteen days after the microbial sample collection, a natural outbreak of *F. columnare* occurred. This resulted in average overall mortality of 4.00 ± 0.01% across the 12 tanks (Isojoki: 2.19 ± 0.004%; Lestijoki: 5.81 ± 0.012%). Mortality was significantly lower in the enriched tanks (GLMM: Χ^2^ = 4.229, *p* = 0.040, rearing), but this was observed mainly in the Isojoki population (GLMM: Χ^2^ = 3.667, *p* = 0.055, rearing $\times$ population interaction). The relationship between proportional mortality and tank biofilm microbial diversity was negative in both Isojoki (Spearman correlation: r_s_ = -0.886, *n* = 6, *p* = 0.019 (OTU number, Fig. 5a), r_s_ = -0.543, *n* = 6, *p* = 0.266 (Shannon diversity)) and Lestijoki populations (r_s_ = -0.943, *n* = 6, *p* = 0.005 (OTU number, Fig. 5b), r_s_ = -0.203, *n* = 6, *p* = 0.700 (Shannon diversity)). The corresponding relationships with the diversity of gut microbiome did not show such consistent patterns (Isojoki: r_s_ = 0.116, *n* = 6, *p* = 0.827 (OTU number), r_s_ = -0.086, *n* = 6, *p* = 0.872 (Shannon diversity); Lestijoki: r_s_ = -0.886, *n* = 6, *p* = 0.019 (OTU number), r_s_ = 0.257, *n* = 6, *p* = 0.623 (Shannon diversity).

## Discussion

Global food production relies on intensive farming. However, diseases represent a major challenge for the farming environments. Treating production animals with pharmaceuticals and chemicals is necessary, but typically result in secondary problems such as resistance evolution and environmental pollution [34, 53]. Therefore, sustainable practices to prevent outbreaks are needed. In aquaculture, environmental enrichment, implemented through additional structures such as stones in rearing tanks, has recently been shown to improve fish survival during disease outbreaks [30, 66]. However, detailed mechanisms underlying the higher fish survival are still unknown. Here, we investigated how microbial diversity and composition in rearing tank biofilm and in fish gut microbiome differed between enriched and standard aquaculture rearing conditions. We focused on three main questions and asked if enriched rearing resulted in higher microbial diversity, if the enriched microbial communities had higher proportion of beneficial microbes, and if the anticipated higher diversity of the microbial community in the enriched rearing environment improved fish survival during a natural pathogen outbreak. We found that enrichment increased microbial diversity and homogeneity in tank biofilm but had a less pronounced effect on gut microbiota. The results also indicated that enriched tanks housed a higher proportion of beneficial microbes and a lower proportion of pathogenic ones. Fish mortality during a bacterial outbreak was also negatively associated with microbial diversity in the tanks, suggesting a protective effect from a more diverse biofilm microbial composition.

The higher microbial diversity observed in the enriched tanks suggests that structures like stones create additional niches for microbes to colonize and persist, and that the higher microbial diversity is mediated also to other surfaces in the tanks. This is reasonable as stones increase the colonization area, represent a natural surface material, and can also change water flow dynamics in the tanks, all of which could potentially have a positive effect on the microbial diversity. However, we did not observe a similar effect of enrichment in gut microbiome, where the microbial diversity was significantly lower and compositionally different compared to tank biofilm. Low gut microbial diversity is common in farmed animals and can be driven by factors such as antimicrobial usage, high sanitation, homogeneous diet, and low contact with natural substrates [1, 14, 83]. Development of gut microbiome composition often also follows different trajectories to water and surface biofilm [23, 79, 87], most likely because of different underlying conditions and selective pressures in gut [39, 72, 77, 80, 83, 86]. Taken together, these results indicate that connection between environmental and gut microbial composition in aquaculture context may not be strong and imply that different types of structural enrichments or alterations in diet are likely to be needed to extend the positive effects of enriched rearing also to gut microbiome.

Interestingly, we also found that the biofilm composition in enriched tanks was more consistent compared to the higher among-tank variation observed in standard tanks. This suggests higher resilience of the enriched microbial communities to chemical-physical fluctuations in the surrounding aquatic environment or, what characterizes the aquaculture realm, to maintenance and cleaning of the tanks. For example, it is possible that stones buffer against mechanical removal of biofilms from tank walls during cleaning (e.g. brushing), therefore maintaining higher consistency in microbial community structure. On the other hand, stones could also contribute to higher inter-individual variation observed in fish gut microbiome in enriched tanks, for example, by allowing some individuals reside next to stones and acquire microbial variation from that micro-habitat. Thus, although proximity and frequent interaction between individuals is known to homogenize microbiomes in a range of invertebrate and vertebrate systems [3, 9, 32, 40, 76, 82, 85], small-scale changes in distribution of individuals could potentially alter these outcomes. The magnitude of microbial variation in relation to enrichments within a school of thousands of fish in a tank, however, remains to be explored. Overall, the above results on microbial diversity and variation suggest that simplified, non-structured environments with high level of hygiene may result in lower microbial diversity and inter-individual variation.

The effects of higher or lower microbial diversity and individual variation on welfare of an organism may depend on which microbial taxa are present in each environment. For example, higher diversity in the interacting microbial community can suppress pathogenic microbes through competition for resources and space, ultimately reducing pathogen presence, abundance and virulence [43, 51, 52]. Here, environmental enrichment decreased bacterial taxa such as *Corynebacteriaceae*, *Flavobacteriaceae* and *Staphylococcaceae*, and increased *Moraxellaceae* in fish gut. Furthermore, *Lactobacilliceae*, which is known for beneficial effects in suppressing pathogen occurrence [19, 55, 59], tended to be more abundant in the enriched fish compared to standard. Whereas *Corynebacteriaceae*, *Flavobacteriaceae* and *Staphylococcaceae* are known to include pathogenic species, the causality between their lower abundance and fish health in enriched environment is difficult to interpret without detailed, species-level genetic analyses. However, the present results suggest that in addition to overall increase in microbial diversity, enrichment can also change the composition of microbial taxa.

Enriched aquaculture rearing is known to improve several fish traits such as growth, tolerance to stress and survival in the wild [22, 28, 57]. One important aspect of wellbeing of fish is the occurrence and severity of diseases, which also has recently been shown to reduce in enriched rearing [28, 66]. While the underlying mechanisms have remained unclear, evidence of enrichments with an established biofilm improving fish survival has indirectly pointed towards microbial communities operating against diseases [30]. In the present study, we found that mortality of fish during a bacterial outbreak in the two fish populations was strongly negatively correlated with the overall microbial diversity in tank biofilm, but not consistently with that of gut microbiome. This provides rare suggestive evidence of external microbial community influencing survival of hosts (see [43, 51, 52] for examples of effects of microbial diversity in other systems). Interestingly, the pattern was evident in each fish population irrespective of their rearing background, suggesting that microbial diversity at the level of individual tanks can potentially influence fish mortality also in standard rearing environment. While the relationships were based on a relatively small number of tanks and need to be corroborated using a broader sampling design, this opens promising avenues for further research on associations between novel rearing methods, microbial composition and epidemiology of aquaculture diseases.

In general, microbial communities could influence disease occurrence through several mechanisms. First, the higher microbial diversity and homogeneity in biofilm of enriched tanks could suppress pathogenic microbes below an epidemiological threshold level required for a disease outbreak. Second, the higher proportion of beneficial microbes (such as lactobacillus) could produce similar outcomes if they outcompeted pathogenic ones for space and resources, or decreased pathogen replication through interference competition. Third, as lower diversity of gut microbiome is known to be linked with increased susceptibility to infections [18, 37], the greater microbial heterogeneity in gut microbiome in enriched tanks could make at least some individuals more resistant to disease [17, 31, 36, 37, 84], again decreasing the likelihood of disease outbreak in a tank. Similarly, more homogenous microbiome in fish in standard tanks could make them more vulnerable to disease establishment and spread [10, 67]. Overall, heterogeneities in microbial diversity and composition in tank biofilm and gut microbiome could provide one explanation for the high variation in disease occurrence and associated mortality typically observed among individual tanks [28, 66].

## Conclusions

Our findings suggest that structural enrichment of aquaculture tanks can enhance microbial diversity in tank biofilms, with potential implications for the heterogeneity of fish gut microbiomes as well. The higher diversity was also associated with lower mortality during a bacterial outbreak, which suggests that measures aiming to promote fish health in aquaculture should consider diversity and structure of microbial communities not just inside the fish, but also in the surrounding tank environment. This has important implications for development of ecologically sustainable rearing practices that aim at minimizing use of antimicrobials and tackling the anticipated increase in diseases occurrence with the ongoing climate change.

## Supplementary Information

Additional file.

## Data Availability

All fastq files obtained from sequencing are publicly available from the NCBI Sequence Read Archive (SRA) database under the accession number PRJNA1108195. The data on fish mortality is available in the JYX Digital Repository of the University of Jyväskylä at following DOI: 10.17011/jyx/dataset/98719.
