# Supplementary material for: Heterogeneity of the rearing environment enhances diversity of microbial communities in intensive farming
Source: Anim Microbiome. 2024 Dec 20;6:75. doi: 10.1186/s42523-024-00359-8 (PMC11662696; doi:10.1186/s42523-024-00359-8)
Supplement: Supplementary file 1 — Additional file. [file 42523_2024_359_MOESM1_ESM.docx]

**Table S1:** Changes of enrichments (water flow dynamics and structures) in enriched tanks during early rearing of brown trout. There were no changes in standard tanks except for the water depth that was changed in the same way as in enriched tanks as the fish grew. Shelters were 25 x 50 cm polystyrene plates supported by metal legs. Each tank had a total of 10 liters of gravel (30-60mm) in two piles.

|  |  |  |  |  |
| --- | --- | --- | --- | --- |
|  |  |  |  |  |


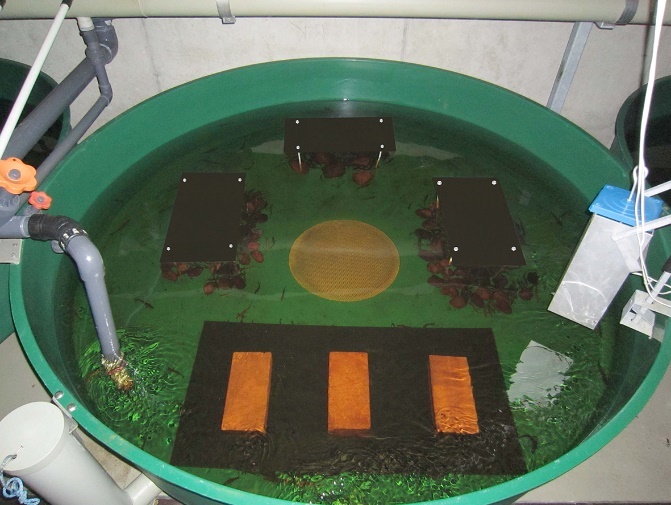

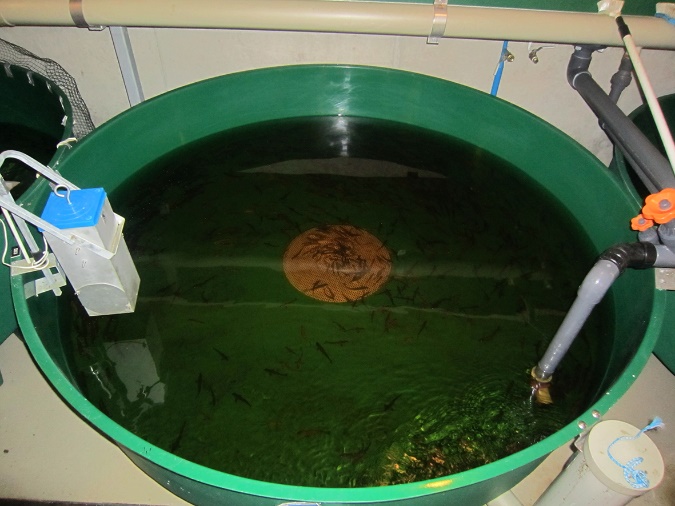


**Figure. S1**: Enriched rearing tank (3.2 m²) with gravel and shelters (left) and standard tanks (right). Photo by Pekka Hyvärinen.


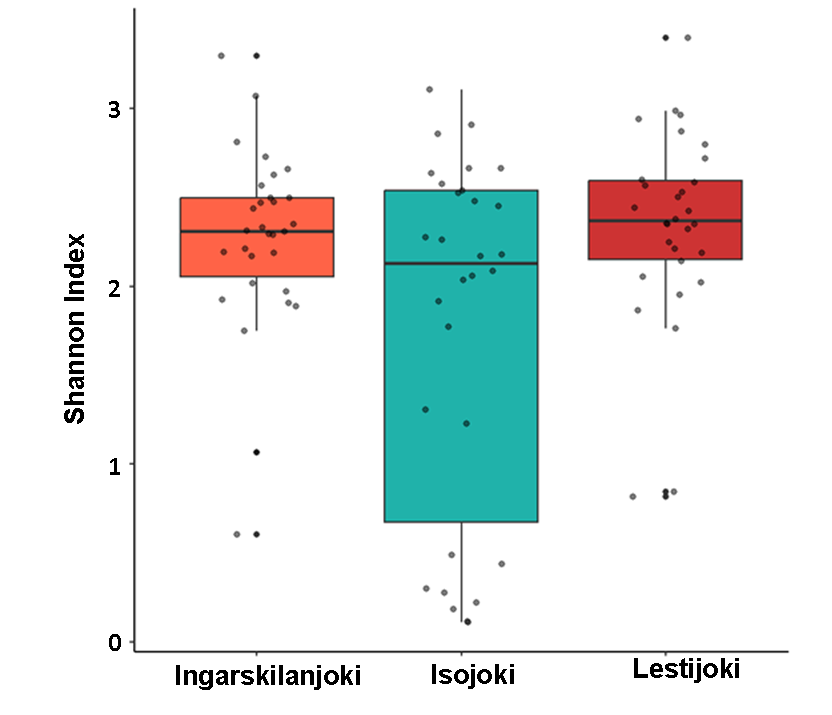


**Figure. S3**: Alpha diversity (Shannon diversity index based on OTU-level analysis) of gut microbial communities in the three populations of brown trout (*Salmo trutta*): River Ingarskilanjoki, Isojoki and Lestijoki.


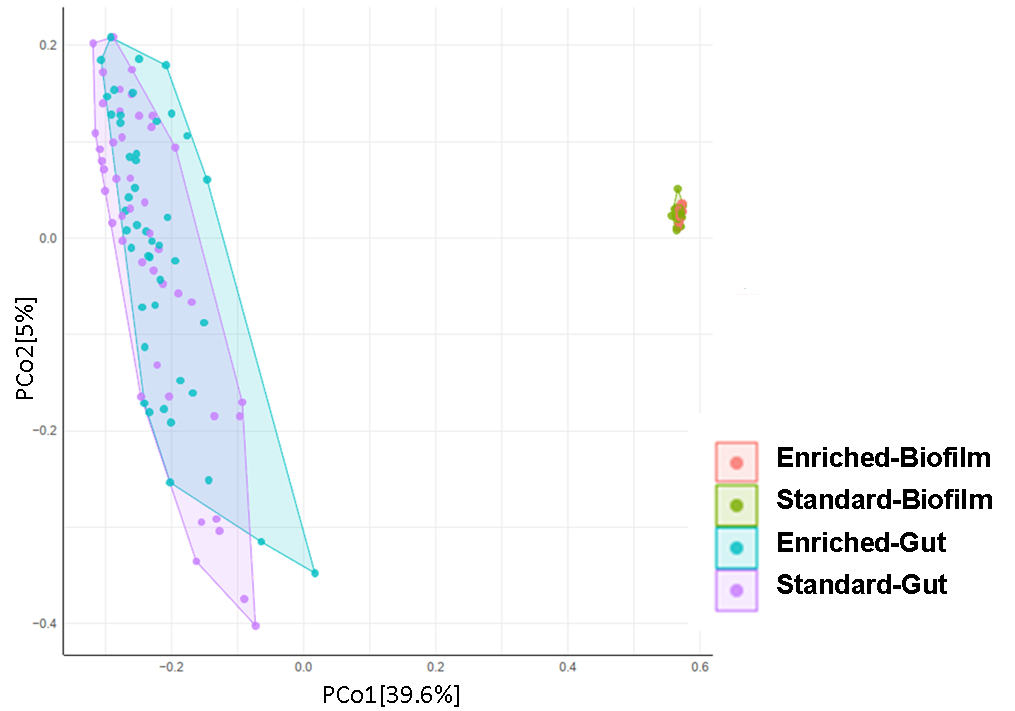


**Figure. S4:** Principal Coordinate Analysis (PCoA) plot on Bray–Curtis dissimilarity distances between tank biofilm and gut microbiome in brown trout (*Salmo trutta*) in enriched and standard rearing treatments. Dots indicate individual samples within the treatment combinations.


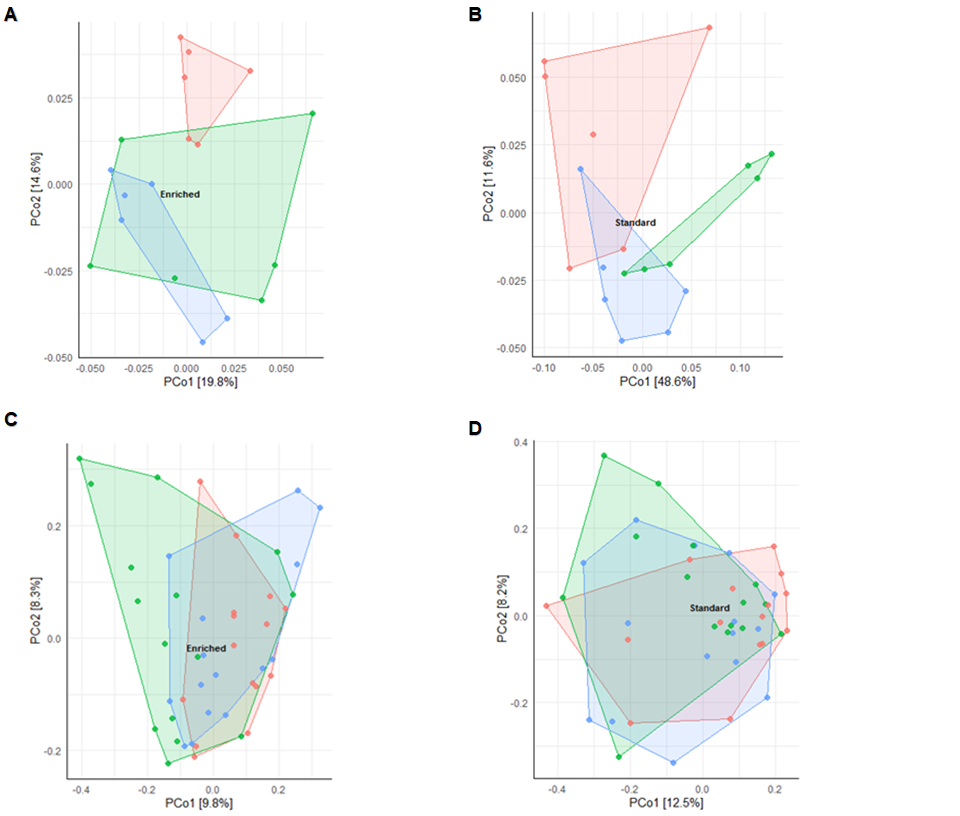


**Figure. S5:** Principal Coordinate Analysis (PCoA) plot on Bray–Curtis dissimilarity distances in microbiome composition in biofilm and gut samples from three populations of brown trout (Salmo trutta) (Rivers Ingarskilanjoki, Isojoki, and Lestijoki). Upper panels (A and B) show biofilm samples, and lower panels (C and D) show gut samples. Panels A and C represent samples raised in enriched tanks (left), while panels B and D represent samples raised in standard tanks (right). Red represents Ingarskilanjoki, green represents Isojoki, and blue represents Lestijoki. Dots indicate individual samples within the treatments.
